# A Novel Design of Grooved Fibers for Fiber-Optic Localized Plasmon Resonance Biosensors

**DOI:** 10.3390/s90806456

**Published:** 2009-08-20

**Authors:** Ching-Te Huang, Chun-Ping Jen, Tzu-Chien Chao, Wei-Te Wu, Wan-Yun Li, Lai-Kwan Chau

**Affiliations:** 1 Department of Mechanical Engineering, National Chung Cheng University, 168, University Rd., Min-Hsiung, Chia Yi, 62102, Taiwan; E-Mails: chingtehuang@gmail.com (C.-T.H.); nick910192@hotmail.com (T.-C.C.); 2 Department of Biomechatronics Engineering, National Pingtung University of Science and Technology, Pingtung 912, Taiwan; E-Mail: weite@mail.npust.edu.tw (W.-T.W.); 3 Department of Chemistry and Biochemistry, National Chung Cheng University, 168, University Rd., Min-Hsiung, Chia Yi, 62102, Taiwan: E-Mails: smilelili72@gmail.com (W.-Y.L.); chelkc@ccu.edu.tw (L.K.C.)

**Keywords:** microfluidic, biosensor, fiber-optic localized plasmon resonance

## Abstract

Bio-molecular recognition is detected by the unique optical properties of self-assembled gold nanoparticles on the unclad portions of an optical fiber whose surfaces have been modified with a receptor. To enhance the performance of the sensing platform, the sensing element is integrated with a microfluidic chip to reduce sample and reagent volume, to shorten response time and analysis time, as well as to increase sensitivity. The main purpose of the present study is to design grooves on the optical fiber for the FO-LPR microfluidic chip and investigate the effect of the groove geometry on the biochemical binding kinetics through simulations. The optical fiber is designed and termed as U-type or D-type based on the shape of the grooves. The numerical results indicate that the design of the D-type fiber exhibits efficient performance on biochemical binding. The grooves designed on the optical fiber also induce chaotic advection to enhance the mixing in the microchannel. The mixing patterns indicate that D-type grooves enhance the mixing more effectively than U-type grooves. D-type fiber with six grooves is the optimum design according to the numerical results. The experimental results show that the D-type fiber could sustain larger elongation than the U-type fiber. Furthermore, this study successfully demonstrates the feasibility of fabricating the grooved optical fibers by the femtosecond laser, and making a transmission-based FO-LPR probe for chemical sensing. The sensor resolution of the sensor implementing the D-type fiber modified by gold nanoparticles was 4.1 × 10^−7^ RIU, which is much more sensitive than that of U-type optical fiber (1.8 × 10^−3^ RIU).

## 1. Introduction

The use of biosensors for the diagnosis of diseases, food testing, and the environmental detection of biological agents has increased dramatically over the past few decades. Recently, a fiber-optic localized surface plasmon resonance (FO-LPR) chemical and biochemical sensing platform for label-free and real-time detection has been developed [1–3]. The unclad portion of an optical fiber is modified with self-assembled gold nanoparticles which are functionalized with a receptor. These nanoparticles characteristically exhibit a strong absorption band that is not present in the spectrum of the bulk metal. The absorption band results when the incident photon frequency is resonant with the collective oscillation of the conduction electrons and is known as the localized plasmon resonance (LPR). The spectral characteristics of the LPR band are highly dependent on the local refractive index of the surrounding medium near the nanoparticle surface, and furthermore, the binding events to those functionalized nanoparticles [4]. However, the signal-to-noise ratio for slide-based LPR sensors is not so high at present, as a result of the low absorbance of the colloidal gold film used. The absorbance of such colloidal gold layers can be enhanced by waveguide sensing which is based on the absorption of the evanescent field via multiple total internal reflections [1]. Here, the sensitivity of the method depends on the length of the waveguide [2]. Hence, with a suitable receptor immobilized at the surface of the gold nanoparticles, the resulting FO-LPR sensor can detect the corresponding analyte even if the analyte is spectroscopically silent in the UV-vis region. Such a FO-LPR sensing platform is sensitive to biomolecular binding events at the pico-molar level [1,2]. In order to enhance the performance of the sensing platform, the sensing element can be integrated with a microfluidic chip to reduce sample and reagent volume, to shorten response time and analysis time, as well as to increase sensitivity [5]. Furthermore, it has been demonstrated that the evanescent absorption coefficient is inversely proportional to the fiber core radius [6,7]. In other words, the smaller the core diameter of the optical fiber, a better performance exhibited by the FO-LPR sensing platform is expected. Thus, a communication grade multimode optical fiber (Corning 62.5/125 Optical Fiber) was employed as the waveguide in the present study. This optical fiber was composed of a silica-based core (62.5 μm in diameter), and cladding and polymeric jacket with outer diameters of 125 and 250 μm, respectively. The number of the total internal reflections per unit length within the optical fiber is expected to increase significantly. However, the major challenge for adopting the optical fiber with such a small diameter is to maintain its mechanical strength during assembling and packaging while the cladding and jacket are removed entirely. To overcome this critical issue, the cladding and polymeric jacket were removed only partially, instead of removing them entirely. The materials wrapped around the core of the optical fiber were ablated using an ultrafast femtosecond laser; then, the core surface was modified with Au nanoparticles and exposed to the sample of analyte. The transparent, hard and brittle material could be effectively machined by the femtosecond laser which induces the non-linear, multi-photon absorption of the material during irradiation [8,9]. The main purpose of the present study was to design grooves on the optical fiber for the FO-LPR microfluidic chip and to investigate the effect of the groove geometry on the biochemical binding kinetics through simulations.

## 2. Designs of the Grooved Optical Fiber

**Figure 1.**
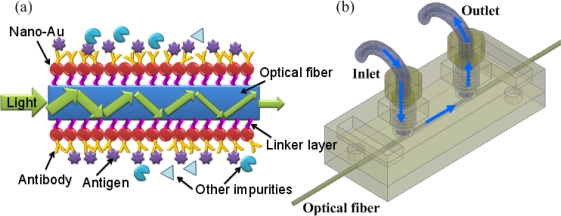
Schematic illustration of the FO-LPR microfluidic chip: (a) detecting principle of the FO-LPR and (b) structure of the chip and illustration of fluidic operation.

The schematic illustration of the FO-LPR microfluidic chip is depicted in Figure 1. Figure 1a shows the detecting principle of the FO-LPR and a sketch of the sensing element. The structure of the chip and the fluidic operation is illustrated in Figure 1b. The solution with analyte was injected into the reaction microchannel and reacted with the receptor coated on the optical fiber. The reaction microchannel was 500 μm high and 500 μm wide with a length of 20 mm. An optical fiber was placed at the center of the microchannel. A gold nanoparticle monolayer was coated on the unclad portion of the optical fiber via an organosilane linker and the gold nanoparticle surface was further functionalized with a receptor [1].

**Figure 2.**
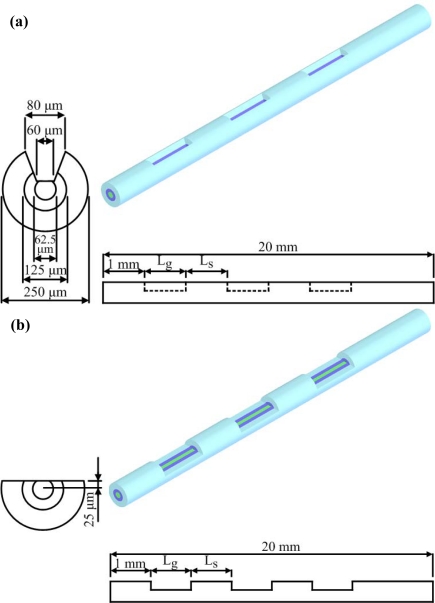
Dimensions and design of the grooved fibers. (a) U-type fiber. (b) D-type fiber.

The value of the Reynolds number for the microchannel of the FO-LPR device was less than ten; therefore, there was laminar flow in the microchannel and molecular diffusion across the channel was slow. To enhance the biochemical binding on the unclad optical fiber, the geometry of the grooved channel was the same as in our previous work [5]. The grooves generate transverse flows in the microchannel [10] and enlarge the probability of analytes getting close to the immobilized receptors. Fabricating grooved microchannels (with a cross section of 500 μm × 500 μm) can be quite complicated. An optical fiber composed of a silica core of 62.5 μm in diameter was employed in the present study. In the original design as reported in the literature [1,2,5], the cladding and jacket layers of a 400 μm core optical fiber were removed entirely. However, the mechanical strength of optical fiber with a core diameter of 62.5 μm, as used herein, was not strong enough to sustain the stresses during assembling and packaging. To avoid fracturing the fiber, the femtosecond laser was used to only partially remove the cladding and polymeric jacket. The present study presents a novel design of grooved fibers which have been integrated into the FO-LPR device. The designs of the grooved optical fibers, as illustrated in Figure 2, are termed as U-type or D-type based on the shape of the grooves.

The length of one groove was L_g_, and L_S_ denotes the space between the grooves, which was fixed at 1 mm. The number of grooves (N_g_) could be varied. In order to compare the performance of U-type and D-type fibers, the total effective area was fixed. Thus, the total length of the grooves was set at 6 mm, i.e., N_g_ × L_g_ equals 6 mm. The mechanical strength of the optical fiber is expected to be improved because of the design of the grooves, and the grooves designed in the optical fiber are also expected to induce chaotic advection to enhance the mixing in the microchannel. The effect of the number of grooves (N_g_) on biochemical binding was then investigated in this simulation study. The enhancement of not only mechanical strength, but also biochemical binding performance by chaotic mixing, is expected in the proposed design.

## 3. Experimental Section

A femtosecond laser micromachining system [9] was used for engraving grooves on the optical fiber. The femtosecond laser was a regenerative amplified mode-locked Ti:sapphire laser with pulse duration ∼120 fs after the compressor, central wavelength 800 nm, repetition rate 1 kHz, and maximum pulse energy of ∼3.5 mJ. The number of laser shots applied to the sample was controlled by an electromechanical shutter. The laser beam was focused onto the fiber by a 10x objective lens (numerical aperture 0.26, M Plan Apo NIR, Mitutoyo) mounted on a Z stage. Grooves under fabrication was translated by a PC controlled X-Y micro-positioning stage with error less than 1 μm. The fabrication process was monitored by a charge-coupled device (CCD). The most prominent features of the femtosecond laser compare with conventional continuous long-pulsed laser are ultra short pulse duration and very strong instantaneous power. According to the above features, the femtosecond laser induces non-linear multi-photon absorption of materials. It can engrave on transparent, hard and brittle materials very precisely without inducing any micro cracks and heat affected zone.

**Figure 3.**
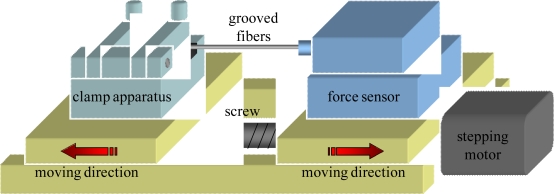
Schematic diagram of the tensile test machine.

The grooved fibers proposed herein is expected to exhibit better perfomance of mechanical strength. The tensile test of the fibers after manufacturing were implemented. Tensile testing is a standard procedure for determining the mechanical properties of materials. A standard tension test machine was set up and shown in Figure 3.

The grooved optical fiber shown is placed in the grips of the testing machine. The grips are driven by stepping motor (the minimum displacement is 1 μm) as well as the screw, hence the load applied by the machine is axial. The testing machine elongates the grooved optical fiber at a slow, constant rate until the grooved optical fiber ruptures. During the test, continuous readings are taken of the applied load and the elongation of the grooved optical fiber. The load-elongation curve diagrams for the optical fibers could be obtained to demonstate their mechanical strength.

**Figure 4.**
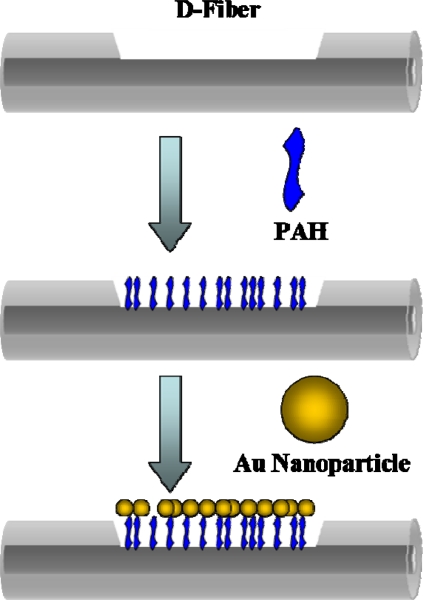
Schematic diagram of the modification of Au nanoparticles on the surface of the grooved optical fiber (D-type fiber was taken as an example).

For the sensor a 62.5/125 multimode all-silica fiber with a 250 μm buffer (Corning) was used. A femtosecond laser micromachining system was used to engrave U or D-shape trenches on the optical fiber. Because of the negative surface charge of gold nanoparticles, the positive charge of poly(allylamine hydrochloride) (PAH) can serve as a linker between the negatively charged silica surface and Au nanoparticles [11–13]. As such, the exposed silica surface in the grooves was then modified with poly(allylamine hydrochloride) by immersing the cleaned grooved optical fibers into vials of 3 mM solution of poly(allylamine hydrochloride). After 15 min, the optical fibers were removed from the solution and rinsed with pure water to remove unbound monomers from the surface. After thorough rinsing, the grooved optical fibers were immersed into the gold nanoparticles solution (prepared based on the procedure of the Natan’s method [14]) for 30 min to modify gold nanoparticles on the surface of the grooves. The optical fibers were then rinsed with pure water to remove unbound gold nanoparticles on the surface. The schematic diagram of the modification of Au nanoparticles was illustrated in Figure 4.

## 4. Numerical Simulations

The mathematical model for mass-influenced binding kinetics has been investigated in the literature [5,15]. Three-dimensional simulations of biochemical binding kinetics were performed using CFD-ACE™ software running on a personal computer, and structured grids were employed to solve the governing equations. The governing equations for the FO-LPR microfluidic chip were the continuity equation, the momentum conservation (Navier-Stokes) equation and the analyte convection-diffusion equation, as well as the surface reaction of a reversible analyte-receptor binding. The dimensionless forms of continuity, momentum and convection-diffusion equations can be expressed as follows:

(1) $$\nabla^{*}\cdot V^{*}=0$$

(2) DV*Dt*=−∇*P*+1Re∇*2V*

(3) $$\frac{{\mathrm{DC}}^{*}}{{\mathrm{Dt}}^{*}}=\frac{1}{\text{Re}\;\mathrm{Sc}}\nabla^{*2}C^{*}$$

where

Re=ρUDhμ, Sc=μρD

C is the concentration of analyte, and *D_h_* and U are the hydraulic diameter of the microfluidic channel and the inlet velocity of the fluid, respectively. Reynolds number (*Re*) is a dimensionless number that characterizes the ratio of inertial forces to viscous forces. It is also used to identify different flow regimes, such as laminar or turbulent flow. The laminar flow occurs at low Reynolds numbers, where viscous forces are dominant. However, turbulent flow occurs at high Reynolds numbers and is dominated by inertial forces, which tend to produce random eddies, vortices and other flow fluctuations. The numerical simulations obtain the pressure, velocity fields and analyte distributions in the microfluidic channel. The Schmidt number (*Sc*) is defined as the kinematic viscosity divided by the diffusivity (*D*) of analyte in the buffer solution. The kinetics of the reversible analyte-receptor binding reaction can be expressed as:

(4) $$A+R\overset{k_{n}}{\underset{k_{d}}{⇄}}\text{AR}$$

where A, R and AR represent concentrations of analyte, receptor binding site and analyte-receptor complex (or bound analyte), respectively. k_a_ and k_d_, respectively, are the association and dissociation rate constants. Laminar flow was set and the temperature effect was omitted in this simulation. Semi Implicit Method for Pressure Linked Equation (SIMPLE) algorithm is initiated by Patankar [16] to solve the above mentioned equations and has been widely applied to Computational Fluid Dynamics (CFD) problems. The SIMPLE-consistent (SIMPLEC) algorithm, which is one of the most popular variants of SIMPLE-family, was proposed to improve the convergence [17]. The SIMPLEC method was adopted for pressure-velocity coupling and all spatial discretizations were performed using the first-order upwind scheme. The simulation was implemented in a transient state. A fixed-velocity condition was set to the boundary condition at the inlet of the FO-LPR microfluidic channel. The boundary condition at the outlet was set at a fixed pressure. The surface of the optical fiber was set as the reactive boundary for biochemical binding. The grid-independency test was done. Therefore, the total number of elements was approximately 50,000 for the cases used in this work. Different numbers of grooves (N_g_) indicate various geometries of computational domains. Three-dimensional simulations of pressure, velocity fields as well as species concentrations including binding reactions are obtained by solving the aforementioned equations. The concentrations of bound analyte on the reaction area are averaged at any instant to illustrate the time histories of binding kinematics.

## 5. Results and Discussion

The parameters of binding kinetics as reported in the literature [18] were adopted in the simulations. The associated and disassociated constants, k_a_ and k_d_, were 57,085 M^−1^s^−1^ and 0.0455 s^−1^, respectively. The surface concentration of the receptor was 9.742 × 10^−8^ mole/m^2^. The physical properties as well as the parameters of binding kinetics are listed in Table 1.

**Figure 5.**
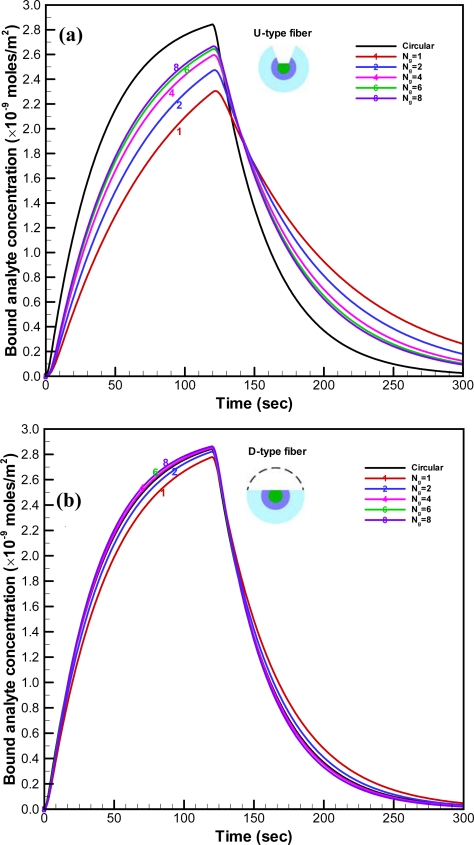
Simulated time histories for the average concentration of bound analyte on (a) the U-type and (b) D type fibers. (a) U-type fiber. (b) D-type fiber.

For the U-type fiber, the simulated time histories for the average concentration of bound analyte on the fiber for different numbers of grooves are plotted in Figure 5a. The concentration of bound analyte increased with the number of grooves (N_g_) in the simulated sensorgram overlays, as shown. However, the increment of the concentration of bound analyte decreased with the number of grooves. Improvement by increasing the number of grooves become smaller when N_g_ is larger than six. The transient concentration of bound analyte when the cladding and jacket layers were removed entirely was simulated for comparison. Since the total effective area should be consistent, the unclad length of the fiber with circular removal was about 3.5 mm. The concentration of bound analyte on the optical fiber with circular removal exhibited the highest values when compared to all the cases of the U-type fiber. When the sample of analyte was injected into the microchannel, the analyte reacted with the binding sites on the unclad portion of the optical fiber. For the optical fiber with circular removal of cladding, the large area with open sites was exposed to the analyte simultaneously. Therefore, the concentration of bound analyte increased much faster than those for the U-type fiber with different number of grooves. For the D-type fiber, the simulated transient histories for the average concentration of bound analyte on the fiber for different numbers of grooves are plotted in Figure 5b. Similar to the simulated results for the U-type fiber, the concentration of bound analyte increased with the number of grooves. Furthermore, the concentration of bound analyte for the D-type fiber with six grooves is almost identical to that with eight grooves. Hence, the D-type fiber with six grooves is the optimum design. Moreover, the concentrations of bound analyte for the D-type fiber are significantly higher than those for the U-type fiber and are comparable to those on the fiber with circular removal of cladding.

**Figure 6.**
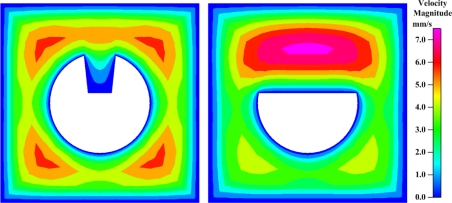
Contours of the flow field in the FO-LPR microfluidic chip with (left) the U-type and (right) the D-type fiber.

**Figure 7.**
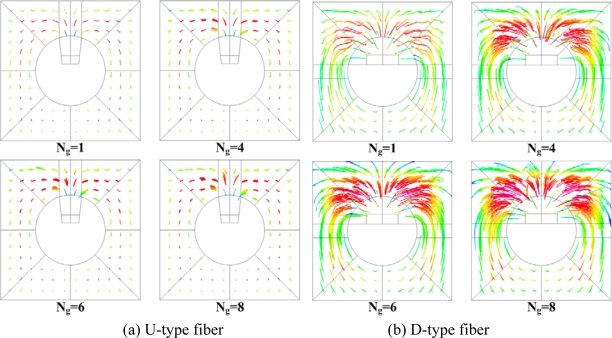
Mixing patterns in the FO-LPR microfluidic chip for (a) the U-type and (b) D-type fiber for numbers of grooves.

The U-shape groove is very narrow; thus, it is not easy for the analyte to get close to the effective area. The contours of the flow field at the center cross-section of the groove in the FO-LPR microfluidic chip with U-type and D-type fibers are depicted in Figure 6. Apparently, since the velocity close to the bottom of U-shape groove is low, most of the binding reaction took place by molecular diffusion rather than advection. The mixing patterns in the FO-LPR chip for U-type and D-type fibers with different number of grooves are shown in Figure 7.

The grooves designed on the optical fiber also induce chaotic advection to enhance the mixing in the microchannel, as shown in this Figure. The mixing patterns point to the fact that D-type grooves enhance the mixing more effectively than U-type grooves. The patterns show that improvement by increasing the number of grooves for U-type fiber become slower. Besides, the mixing patterns for the D-type fiber indicate that the degrees of chaos in the microfluidic channels for N_g_ equals 4, 6 or 8 are close, which is consistent with the results in Figure 5b. The optimum performance of biochemical binding for the D-type fiber is close to that for the optical fiber with circular removal of cladding, as shown in Figure 5b. The peak value of the concentration of bound analyte on the D-type fiber with six grooves is 0.7% higher than that of the optical fiber with circular removal. The performance of the D-type fiber with six grooves is as good as that of the fiber with circular removal. However, the D-type fiber with six grooves remains the optimum design due to the increased fragility of the fiber with increased number of grooves.

**Figure 8.**
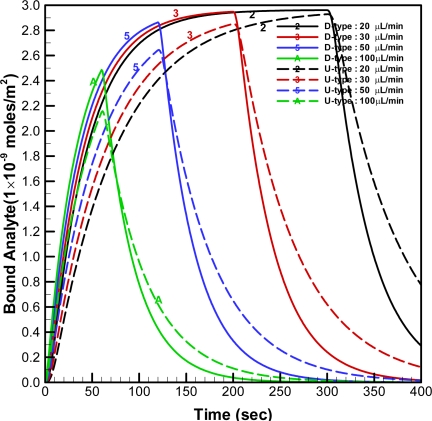
Simulated time histories for the average concentration of bound analyte on the U-type and D-type optical fibers (N_g_ = 6) with different injected flow-rates when the injected volume was fixed at 100 μL.

When the injected volume was fixed at 100 μL, the simulated time histories for the average concentration of bound analyte on the U-type and D-type optical fibers (N_g_ = 6) with different injected flow-rates are shown in Figure 8.

The concentration of bound analyte for the higher injected flow-rate increases faster than that for the lower injected flow-rate, as shown in this figure. However, the duration of the free analyte staying in the channel becomes shorter when the higher injected flow-rate is applied. Therefore, the bound analyte is not allowed to reach equilibrium concentration in the cases with the higher injected flow-rates (e.g. when the flow-rate was 100 μL/min). When the flow rate is 50 μL/min, the concentration of bound analyte on the D-type fiber reaches 97% of the equilibrium concentration; however, that on the U-type fiber is only 89% of the equilibrium concentration. The response time may be quantitatively defined as the required time to reach 95% of the equilibrium concentration when the flowrate is 20 μL/min. Therefore, the response times with the D-type and U-type are approximate 122 and 206 seconds, respectively. This indicates the response time with the D-type fiber is faster than that with the U-type fiber.

**Figure 9.**
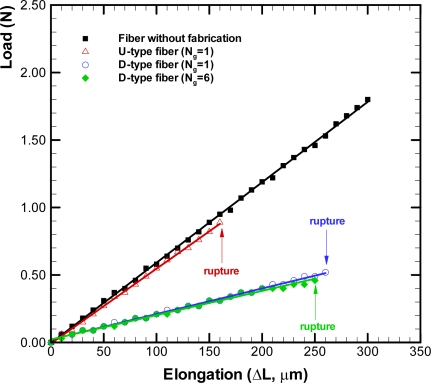
The load-elongation curve diagrams for optical fibers with different types of grooves.

The load-elongation curve diagrams for optical fibers with different types of grooves are depicted in Figure 9. The symbols represent the experimental data and linear least square fitting curves of these data are also plotted herein. The curve for the U-type fiber with one groove is close to that for the fiber without fabrication. However, the U-type fiber ruptured when its elongation was beyond 160 μm, which was only half of that for a raw fiber. The curves of load-elongation for the D-type fibers with one and six grooves are close and their elongations can be about 250 μm. The D-type fibers with one and six grooves ruptured when their elongation were beyond 260 μm and 250 μm, respectively. The elongation for D-type fibers apparently can be larger than that for the U-type fiber. Moreover, the slope of the curve for the D-type fiber is smaller than that for the U-type as well as raw fibers. It indicates that the D-type fibers exhibit larger elongation than the U-type fiber when the same loading is applied. The experimental results show that the D-types fiber can sustain larger elongation than the U-type fiber. Moreover, the effect of increase of the number of grooves in the D-type fiber is almost insignificant as shown in the load-elongation curve diagrams.

**Figure 10.**
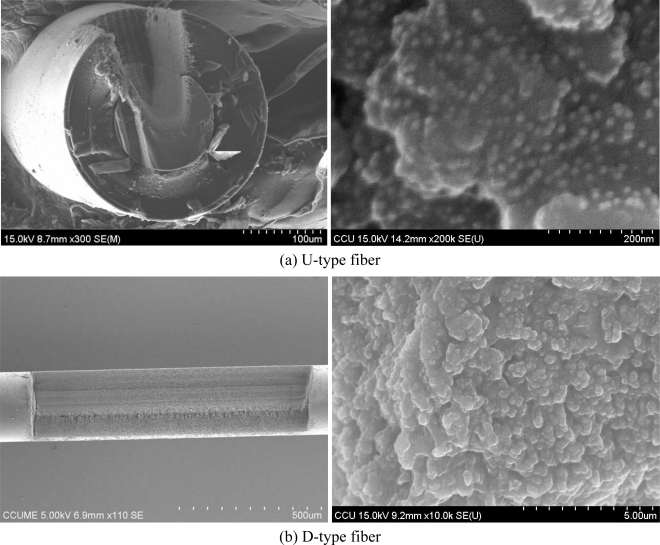
SEM images of (left) the optical fiber fabricated by the femtosecond laser and (right) its exposed surface after modification by gold nanoparticles for (a) U-type and (b) D-type fibers.

Figure 10 shows the SEM images of the optical fibers fabricated by the femtosecond laser and its exposed surface after modification by gold nanoparticles for the U-type and D-type fibers. The dimensions of the U-shape groove, illustrated in Figure 10a (left), were 100 μm in depth measured from the surface of the polymer jacket layer, 80 μm in width in the jacket layer, 60 μm in width in the cladding layer and the total length is 6 mm (N_g_ = 1). It indicates that the core of the fiber has been exposed and the remaining jacket layer has provided enough mechanical strength for further processing according to the aforementioned results. The SEM image of the exposed surface of the U-type fiber after gold nanoparticles-modification is revealed in Figure 10a (right). Apparently, Au nanoparticles are distributed on the surface uniformly. However, it should be noted the SEM image could be taken only at a silica surface close to the jacket, due to the difficulty of imaging bottom silica surface by SEM. The SEM images of the D-type optical fiber fabricated by the femtosecond laser and its exposed surface after modification by gold nanoparticles are presented in Figure 10b. The dimensions of the D-shape groove shown in Figure 10b were 100 μm in depth measured from the surface of the polymer jacket layer, and the total length is 6 mm (N_g_ = 1). It also indicates that gold nanoparticles have been successfully immobilized on the exposed surface of the fiber.

The fiber-optic sensing system set up to measure the transmission power of the sensor was reported in our previous work [9]. This system was consisted of a function generator, a LED light source (λ = 530 nm), a sensing grooved fiber modified with Au-nanoparticles, a microfluidic chip, a photodiode, a lock-in amplifier and a computer for data acquisition. The abilities of the Au-modified grooved optical fibers with one groove to detect changes in the surrounding refractive index were investigated. The surrounding refractive index was controlled by preparing sucrose solutions with various concentrations [19]. The refractive indexes of sucrose solutions were prepared in the range of 1.333 to 1.403. For the U-type optical fiber (N_g_ = 1), a plot of the transmission power as a function of the refractive index was linear (*R* = 0.9998). The sensor resolution by transmission power interrogation (sensor resolution = 3 σ/*m*, σ = standard deviation of *I* in measuring the blank, *m* = slope) was 1.8 × 10^−3^ RIU. For the D-type optical fiber (N_g_ = 1), a plot of the transmission power as a function of the refractive index was also linear (*R* = 0.9983). However, the sensor resolution by the D-type fiber was determined to be 4.1 × 10^−7^ RIU, which is significantly better than that obtained by the U-type fiber. The reason for such a big improvement is not clear, and is likely caused by a significantly lower surface coverage of gold nanoparticles on the U-type fiber core as a result of poor mass transfer of gold nanoparticles and/or linker molecules to the fiber core surface.

## 6. Conclusions

In this study, the numerical simulation of biochemical binding kinetics of the FO-LPR microfluidic chip with grooved optical fibers was successfully performed. The sensing element of the FO-LPR sensing platform was integrated with the microfluidic chip to reduce sample and reagent volume, to shorten both response and analysis time, as well as to increase sensitivity. The optical fibers were designed and termed as U-type or D-type based on the shape of the grooves. The U-shape groove was so narrow that it was not easy for the analyte to get close to the effective area. For the optical fiber with circular removal of cladding, the large area with open sites was exposed to the analyte simultaneously. Therefore, the concentration of bound analyte increased much faster than that for the U-type fiber. However, the mechanical strength of the optical fiber with a core diameter of 62.5 μm, as used herein, was not strong enough to sustain the stresses during assembling and packaging. The concentration of bound analyte increases with the number of grooves (N_g_) in the simulated sensorgram overlays. The numerical results indicate that the design of the D-type fiber has exhibited efficient performance on biochemical binding. The grooves designed on the optical fiber can also induce chaotic advection to enhance the mixing in the microchannel. The mixing patterns indicate that the D-type grooves enhance the mixing more effectively than the U-type grooves. The optimum performance of biochemical binding for the D-type fiber (N_g_ = 6) is very close to that for the optical fiber with circular removal of cladding. The experimental results indicate that the D-type fiber performs better than the U-type fiber in terms of the mechanical property. Furthermore, the present study successfully demonstrates the feasibility of fabricating the grooved optical fibers by the femtosecond laser, and making a transmission-based FO-LPR probe for chemical sensing. The sensor resolution of the gold nanoparticles-modified D-type optical fiber by transmission power interrogation is 4.1 × 10^−7^ RIU, which is much more sensitive than that of U-type optical fiber (1.8 × 10^−3^ RIU).

## Figures and Tables

**Table 1.** Parameters and physical properties used in the simulations.

**Properties**	**Values**

Association rate constant, k_a_	57,085 M^−1^s^−1^
Dissociation rate constant, k_d_	0.0455 s^−1^
Inlet analyte concentration, C_0_	25 nM
Maximum possible surface analyte concentration, P_s_	9.742 × 10^−8^ mole/m^2^
Density of the sample, *ρ*	997 kg/m^3^
Viscosity of the sample, *ν*	0.86 × 10^−6^ m^2^/s
Diffusivity of analyte, *D*	10^−10^ m^2^/ s
Total injected volume of sample	100 μL
Inlet volumetric flow rate, Q	50 μL/min
